# Correction to “Reliability, Sensitivity, and Predictive Value of fMRI During Multiple Object Tracking as a Marker of Cognitive‐Training Gain in Combination With tDCS in Stroke Survivors”

**DOI:** 10.1002/hbm.70439

**Published:** 2025-12-15

Kolskår, K. K., G. Richard, D. Alnæs, et al. “Reliability, Sensitivity, and Predictive Value of fMRI During Multiple Object Tracking as a Marker of Cognitive‐Training Gain in Combination With tDCS in Stroke Survivors.” *Human Brain Mapping* 42, no. 4: 1167–1181. https://doi.org/10.1002/hbm.25284.

We discovered that the PCA‐derived Cogmed change score had a shift in the sign such that the direction change it indicated was inverted. We have corrected this by multiplying the PCA scores by −1. This adjustment leaves all *p*‐values and significance conclusions unaffected and thus does not alter the main finding of no significant associations between tDCS group, baseline cognitive level, or fMRI activation and the Cogmed change score.

We used the *mvoutlier* package to identify potential outliers before applying PCA to generate the Cogmed change score. Based on this test, we excluded two Cogmed subtests from further analyses. We have since discovered that the assumption of multivariate normality required for *mvoutlier* was violated (Henze–Zirkler test: HZ = 9.692, *p* < 0.001). To assess the impact of removal of these two sub‐tests on the results, we have added new analyses with these two sub‐tests. Accordingly, the updated supplementary tables now report results based on both the original six‐subtest PCA solution and an alternative eight‐subtest solution that includes the two previously excluded subtests. We found only minor differences in the associations involving the Cogmed change score, lesion characteristics, as well as Cabpad baseline performance, and the overall pattern of results remains consistent with those originally reported. For the Cogmed change score–fMRI associations, we again found no significant relationships, in line with the original findings. For transparency and reproducibility, we have made the statistical maps underlying the Cogmed–fMRI association analyses available on OSF (https://doi.org/10.17605/OSF.IO/NDPUZ).

Additionally, in Supplementary Table S5 and Supplementary Figure S6, the variable *number of lesions* was erroneously used in place of the *Cogmed change score*. Correcting this did not alter any significant associations or conclusions. Updated versions of the affected table and figure including the Cogmed change score for both eight and six Cogmed‐based PCA‐derived scores have been corrected in the online file.

Following review of contributions, Henrik Formoe has been added as a co‐author. He has contributed substantially to the identification of these errors, reanalysis of data as well as interpretation, and thereby meeting the criteria for authorship according to journal and ICMJE guidelines.

The corrected author list is:

Kolskår, K. K., Richard, G., Alnæs, D., Dørum, E. S., Sanders, A. ‐M., Ulrichsen, K. M., Monereo Sánchez, J., Ihle‐Hansen, H., **Formoe, H**., Nordvik, J. E., and Westlye, L. T.

All authors, including the newly added author, have reviewed and approved this correction.

The online version of the author‐list has been corrected accordingly.

We apologize for this error.

Corrected text:

Section 2.9.2 Cogmed individual trajectories

Original text: “We used the first factor from the beta PCA as a Cogmed change score.”

Corrected text:

“We used the first factor from the beta PCA as a Cogmed change score. For interpretability, this component was multiplied by −1 so that higher values indicate larger training gains.”

Section 3.1 Cogmed performance

Original text: “We found no significant correlation between Cogmed average performance and Cogmed change score (r = 0.17, t = 1.29, p = 0.2). We found a significant association between lesion volume and Cogmed change score (t − 2.5, p = 0.017), indicating a negative correlation between lesion volume and training response, which did not retain significance after removal of one outlier (t = −0.58, p = 0.563). See Table S3 for corresponding statistics. Furthermore, results revealed no significant correlations between time since injury and Cogmed average performance (r = −0.19, t = −1.38, p = 0.173) nor Cogmed change score (−0.19, t = −1.35, p = 0.181).”

Corrected text: “We found no significant correlation between Cogmed average performance and Cogmed change score (*r =* −0.17, *t* = −1.29, *p* = 0.2). We found a significant association between lesion volume and Cogmed change score for both six (*t* = 2.5, *p* = 0.017) and eight (*t* = 3.2, *p* = 0.003) subtest‐based Cogmed change score, indicating a positive correlation between lesion volume and training response, which did not retain significance after removal of one outlier (six subtests: *t* = 0.58, *p* = 0.563, eight subtests: *t* = 1.69, *p* = 0.098). See Table S3 for corresponding statistics. Furthermore, results revealed no significant correlations between time since injury and Cogmed average performance (*r* = −0.19, *t* = −1.38, *p* = 0.173) nor Cogmed change score (0.19, *t* = 1.35, *p* = 0.181).”

The supporting information for Tables S3 and S5 and Figure S6 have been corrected online.

